# Exploration on the Influencing Factors of Overseas Students' Online Chinese Learning Intention During the Epidemic Period

**DOI:** 10.3389/fpsyg.2022.907965

**Published:** 2022-05-25

**Authors:** Xiaoyu Zou

**Affiliations:** International College of Chinese Studies, Fujian Normal University, Fuzhou, China

**Keywords:** online learning, willingness to learn, influencing factors, structural equation modeling, COVID-19 pandemic

## Abstract

In the information age, online teaching has become an essential field of educational development. The work aims to analyze the factors influencing learning intention of Overseas Students (OSs) during the Coronavirus Disease 2019 (COVID-19). The work adopts the method of Unified Theory of Acceptance and Use of Technology (UTAUT), and implements the influencing factor model of open source software online Chinese learning (OCL). Specifically, the OSs of five colleges and universities in Fuzhou are taken as the research objects. The data is collected through questionnaire survey (QS) and Spss25.0 to analyze the reliability and validity of the data, and Amos23.0 is used to validate the model. The results present that autonomy and self-discipline have become the necessary factors affecting open source software OCL, and personal interests and hobbies are the main factors affecting open source software OCL. Meantime, perceived interest, self-learning management, effort expectation, community influence, performance expectation, and promotion conditions have a significant positive impact on the willingness to promote Chinese learning, and their influence degree increases in turn. The conclusion can provide a novel online teaching and learning strategy for open source software under the COVID-19 situation.

## Introduction

Due to the ongoing Corona Virus 2019 (COVID-19) pandemic, Online Teaching and Learning (OTL) has become a general trend in most countries across the world (Spitzer et al., 2021). Back in early 2020, the COVID-19 outbreak has hindered many students from going to school for normal educational courses. To that end, the Chinese Ministry of Education (MoE) has issued some policy packages to encourage OTL, including “Classes suspended but learning continues.” Overnight, OTL has gone viral in China, with many effective measures being put forward (Agyeiwaah et al., 2021; Al-Nasa'h et al., 2021). As of February 2020, the MoE has organized 22 OTL platforms to open free Online Courses (OLC)s for Chinese College Students (CCSs). Remarkably, the “Chinese University MOOC” website provides nearly 8,000 high-quality OLCs. To help CCSs learn and study normally during the COVID-19 pandemic, Chinese higher institutions have dedicated immensely to high-quality OLC development tailored to CCSs' practical needs. Statistics show that China has the most vigorous OTL implementation during the COVID-19 crisis, so OTL has seen unprecedented popularity and technical maturity. However, whether to adhere to OTL during and after school suspension remains investigated.

The introduction of OLC has revolutionized the traditional teaching model, the success and failure of which largely depends on students' and teachers' skills and dexterity to manipulate OTL-oriented Applications (APPs) (Jacqmin, 2021; Muflih et al., 2021). Meanwhile, OTL requires novel teaching methods and proactive learning attitudes, which run throughout the whole OTL process (Maqableh and Alia, 2021). To date, there has been extensive research on OTL. Some put forward the hypothesis of influencing factors of College Students' willingness to use mobile OTL platform based on User-Perceived Value (UPV) theory and verified the hypothesis by Questionnaire Survey (QS) and Structural Equation Modeling (SEM). The results show that the designed QS has good reliability and validity. Meanwhile, the network's exploratory learning and good reputation positively impact college students' perception of the Ease of Use (EoU) and usefulness of the mobile OTL platform. Perceived usefulness has a significant direct and positive impact on their willingness to use the online education platform.

Willingness To Learn (WTL), a key factor determining the results of Chinese learning, can arouse learning activities and learning behaviors (Catalano et al., 2021; Liu et al., 2021). At present, there are few studies on WTL, and they are basically the summary of the education reform experience of education suppliers. Therefore, there is a lack of empirical investigation on the online education demanders' [namely Overseas Student (OS)] Chinese WTL. With the push of the Belt and Road initiative, OSs are increasing. Thus, Chinese teaching has become critical for OSs. Thereupon, the present work studies the online Chinese WTL in five universities in Fuzhou and collects and discusses the factors controlling the OSs' Chinese WTL, which provides a reference for developing Chinese OTL strategies for OSs under the COVID-19 pandemic.To sum up, with regard to the analysis of foreign students' Online Chinese learning (OCL) intention, the advantage of the current studies is that a large number of learning data of foreign students can be collected through questionnaire survey and SEM to model and analyze the degree of learning intention. However, the disadvantage of these studies is that the effect of empirical test is poor. Therefore, the model needs to be further improved and optimized.

WTL of five universities in Fuzhou is discussed. The main innovation is to collect and discuss the important factors affecting foreign students' Chinese learning intention. First, the background of the COVID-19 and the traditional teaching mode of OCL are explored. Through the study of OCL strategies and characteristics, the factors of satisfaction of OCL for foreign students are modeled, and the OCL-oriented operating system is analyzed as a model. Through the design and distribution of questionnaires, the reliability of the sample is tested and the results of the model fitting are obtained. The results reveal that the standardized coefficients of Overseas Students' Facilitating Conditions (OSFC), Overseas Students' Self-learning Management (OSSM), and Overseas Students' Perceived Interest (OSPI) for Overseas students' willingness to learn Chinese Online (OSLW) are 0.156, 0.254, and 0.289, respectively. The work provides a specific experimental reference for the development of open source software under the leadership of the COVID-19 outbreak and the promotion of OCL efficiency.

## Characteristics of OTL

Different teaching methods and learning modes have different characteristics. In particular, the traditional class teaching system has been proposed to meet the needs of large-scale talent training. Still, the new *University Geography* Education *Resource Bank* provides a novel approach to breaking the barriers of economic and geography education resources. Meanwhile, with the advent of the learning society, e-learning has gained more momentum, and significant changes have taken place in teaching modes and even classroom time allocations (Moura et al., 2021; Yu, 2022). Additionally, the popularity of the Internet also has a significant impact on learners' physical, mental, and family conditions, rendering some unique characteristics for OTL.

During the COVID-19 pandemic, OTL is still based on classes, teaching materials, and timetables. However, the teaching location is not fixed due to the geographic separations, the teaching content will be reduced, and the interaction between teachers and students will be weakened. The result is it alters the platform on which the OLC depends, the concept of teaching basis, students' participation, and sense of experience (Binali et al., 2021; Thepwongsa et al., 2021). Generally speaking, under the background of the COVID-19 pandemic, the OTL features a traditional classroom, MOOC, and mixed teaching. At the same time, OTL's characteristics might differ due to the audiences and teaching motives. Therefore, there is a need to consider OTL beyond single traditional teaching, online learning, or mixed teaching modes but comprehensively analyze its characteristics. In other words, to explore the factors of online learners' WTL, there is a need for educators to consider both the universality and particularity of OTL.

## Online Chinese Learning (OCL)-Oriented Learning Satisfaction (LS) Factor Modeling

**Figure 1:**
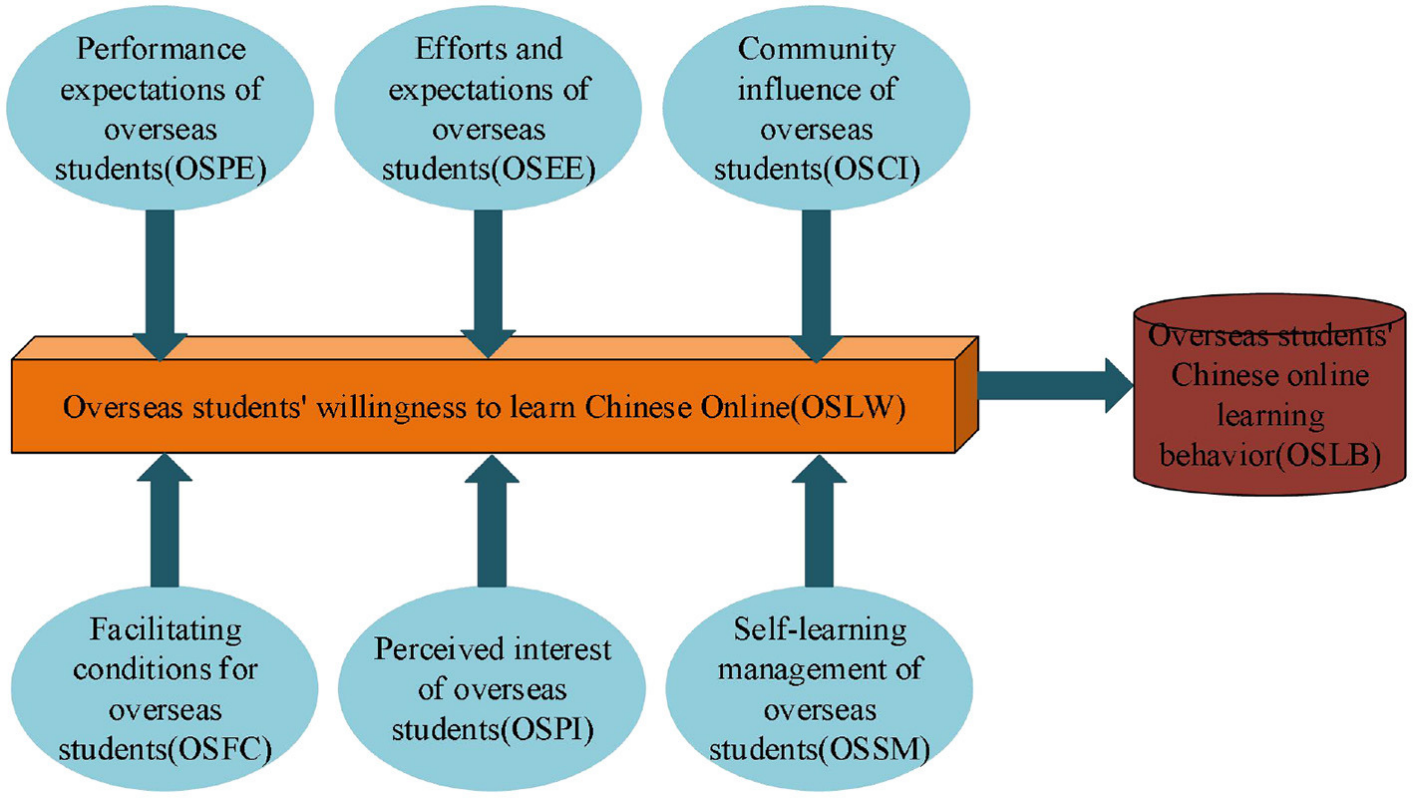
OCL-oriented OS WTL factor model.

Based on the Unified Theory of Acceptance and Use of Technology (UTAUT), this section implements the OCL-oriented OS WTL factor model, as shown in Figure 1.

Generally, UTAUT is believed to have the highest power in understanding and explaining Behavioral Intention (BI) and usage behavior, but it has some limitations. Thus, this section does not employ UTAUT to model the OS WTL factors directly but adds new variables concerning the COVID-19 pandemic and the OS characteristics. In so doing, OS WTL for OCL can be better explained during the COVID-19 pandemic by introducing two independent variables: Self-Learning Management (SLM) and Perceived Interest (PI). For example, studies have shown that learners with strong SLM abilities have stronger online WTL (Theobald, 2021). Meanwhile, others believe that SLM ability directly affects online learning, and consciousness and self-discipline are also important (Hong et al., 2021). More precisely, PI refers to the learners' pleasantness during online learning, and the flexibility and interactivity of online learning can better stimulate students' learning interest (Apostolou, 2021; Nuutila et al., 2021; Oppermann and Lazarides, 2021). Additionally, most OSs lack learning motivation for Chinese and might have difficulty completing the whole course. Thus, the interest of the course is crucial to stimulate learning motivation.

### Research Hypothesis

Firstly, in the UTAUT model, performance expectation refers to the extent to which users believe that technology can help them improve their work performance (Erjavec and Manfreda, 2021). It is the main factor affecting users' online learning. The current work believes that when OSs feel that online learning can improve their Chinese level, their WTL gets stronger. Secondly, effort expectation in UTAUT is user perception of how convenient technological usage is (Andrews et al., 2021). Many studies reveal that effort expectation directly or indirectly impacts online learning BI. The present work holds that when learners can use online learning more efficiently and skillfully, they have a stronger WTL. Thirdly, community influence in UTAUT is the users' perception surrounding groups' attitudes toward a technology (Jadil et al., 2021). Relevant studies have uncovered that community influence directly or indirectly impacts online learners' Willingness To Use (WTU) (Akinnuwesi et al., 2021). This study contends that external factors, such as school fellows, teachers, and parents, significantly impact online learners' WTU. Fourthly, facilitating conditions in UTAUT are objective factors making technology easier (Bu et al., 2021). Users who feel objective conditions are easy to meet will exert higher BI (Ayuning Budi et al., 2021). Thus, if the online learners feel objective conditions for using OTL platforms are easy to meet, their WTU will be stronger (Ye et al., 2020). On the other hand, SLM is an individual's perception of the degree of self-discipline and autonomous learning. Particularly, since online learning features the “three separations” characteristics, learners are required to have better SLM ability. Research shows that a lack of SLM ability will impact the use of OTL platforms (Vinette and Bilodeau, 2021). The present work argues that students with solid SLM ability have a strong online WTL. Meanwhile, the higher the PI is, the stronger learners' WTU to use OTL platforms. Lastly, BI is the degree to which OSs are willing, intend, or use OTL platforms again.

Based on the above analysis, the research hypotheses are put forward in Table 1. After the questionnaire survey, the fitting evaluation model is implemented by using the collected data, and these hypotheses are experimentally verified:

**Table 1:** Research hypotheses.

**Label**	**Research hypotheses**
H1	OSs' Performance Expectation (OSPE) has a significant positive impact on OSs' WTL (OSTL) for OCL.
H2	OSs' Effort Expectation (OSEE) has a significant positive impact on OSTL for OCL.
H3	OSs' Community Influence (OSCF) has a significant positive impact on OSTL for OCL.
H4	OSs' Facilitating Condition (OSFC) has a significant positive impact on OSTL for OCL.
H5	OSs' SLM (OSSM) has a significant positive impact on OSTL for OCL.
H6	PI has a significant positive impact on OSTL for OCL.
H7	BI has a significant positive impact on OSLB for OCL.

Based on the above analysis, Table 1 proposes the research hypotheses:

As Table 1 shows, H1 is the positive impact of OSPE on OSTL, H2 is the positive impact of OSEE on OSTL, H3 is the positive impact of OSCI on OSTL, H4 is the positive impact of OSFC on OSTL, H5 is the positive impact of OSSM on OSTL, H6 is the positive impact of OSTL on OCL, H7 is the positive impact of OSLB on OCL. The hypothesis is verified by the fitting evaluation model, and the estimation result is the hypothesis that has passed the test.

### Design of Questionnaire Survey (QS)

Next, this section collects data through QS to verify the proposed model. Before QS design, the existing scales are sorted out to determine the measurement items of each dimension based on the situation of the present work. Meanwhile, experts are invited to discuss and modify the QS items. Then, the designed QS is pre-tested and adjusted accordingly. Finally, the experiment designs an OSs' OCL-oriented WTL influencing factors QS.

The QS comprises a personal information survey and an influencing factors survey. The options adopt Likert level 5 items: 1-disagree and 5-fully agree. The theoretical model measures the eight variables: OSPE, OSEE, OSCI, OSFC, OSLM, OSPI, OSLW, and the OSLB. Table 2 lists the set measurement items for the specific variables:

**Table 2:** Measurement items of variables.

**Variable**	**Identifier**	**Item**
OSPE	OSPE1	I think OCL can improve my Chinese learning efficiency.
	OSPE2	I think OCL can help preview and review Chinese.
	OSPE3	I think OCL can meet my needs for learning Chinese.
	OSPE4	I think more Chinese learning resources can be accessed through OCL.
OSEE	OSEE1	I think the terminal equipment for OCL is relatively easy to use.
	OSEE2	I think I can quickly adapt to the way of OCL.
	OSEE3	I find it easy to do what I want to do with OCL.
	OSEE4	I can absorb and understand the knowledge points mentioned in OCL.
OSCI	OSCI1	If my relatives or friends let me learn Chinese online, I will be willing to use it.
	OSCI2	If all the students around me learn Chinese online, I will be willing to use it.
	OSCI3	If teachers or classmates recommend that I learn Chinese online, I will be willing to use it.
	OSCI4	If I advocate OCL, I will be willing to use it.
OSFC	OSFC1	I have the software and hardware conditions for OCL.
	OSFC2	I have the necessary knowledge of OCL.
	OSFC3	If someone can help me solve the difficulties in OCL, I will be willing to use it.
	OSFC4	If OCL can complement the advantages of traditional offline learning, I am willing to use it.
OSSM	OSSM1	I can set my own goals for OCL.
	OSSM2	I can reasonably arrange OCL time.
	OSSM3	I have good self-discipline and will not be affected by other social media.
	OSSM4	After a stage of OCL, I will conduct a self-evaluation.
OSPI	OSPI1	Online discussion and interaction can increase the interest of OCL.
	OSPI2	The content of OCL is more vivid than the traditional classroom.
	OSPI3	I can choose my favorite teacher to teach Chinese online, which makes me very happy.
OSLW	OSLW1	If conditions permit, I prefer to choose OCL.
	OSLW2	I am willing to continue OCL in the future.
	OSLW3	I would like to recommend OCL to my friends and classmates.
OSLB	OSLB1	I will start OCL.
	OSLB2	I will continue OCL.
	OSLB3	I will recommend it to the people around me to learn Chinese online.

### QS Distribution and Recovery

This section recruits OSs from five higher institutions in Fuzhou as research subjects because these students have experience of the COVID-19 epidemic and online learning. Thus, sample selection is representative. Then, the Questionnaire Star generates the electronic and word versions of the QS, distributed online. Totally, 500 QSs are distributed, with 100 pieces for each school, and 480 QSs are recovered. After removing invalid QS, such as incomplete answers and inconsistent answers, the 438 pieces are effective, with an effective rate of 91.25%. Meanwhile, there are 29 measurement items, and the effective QSs are over 290, so SEM can analyze the data.

## Result Analysis

### Descriptive Statistics

**Figure 2:**
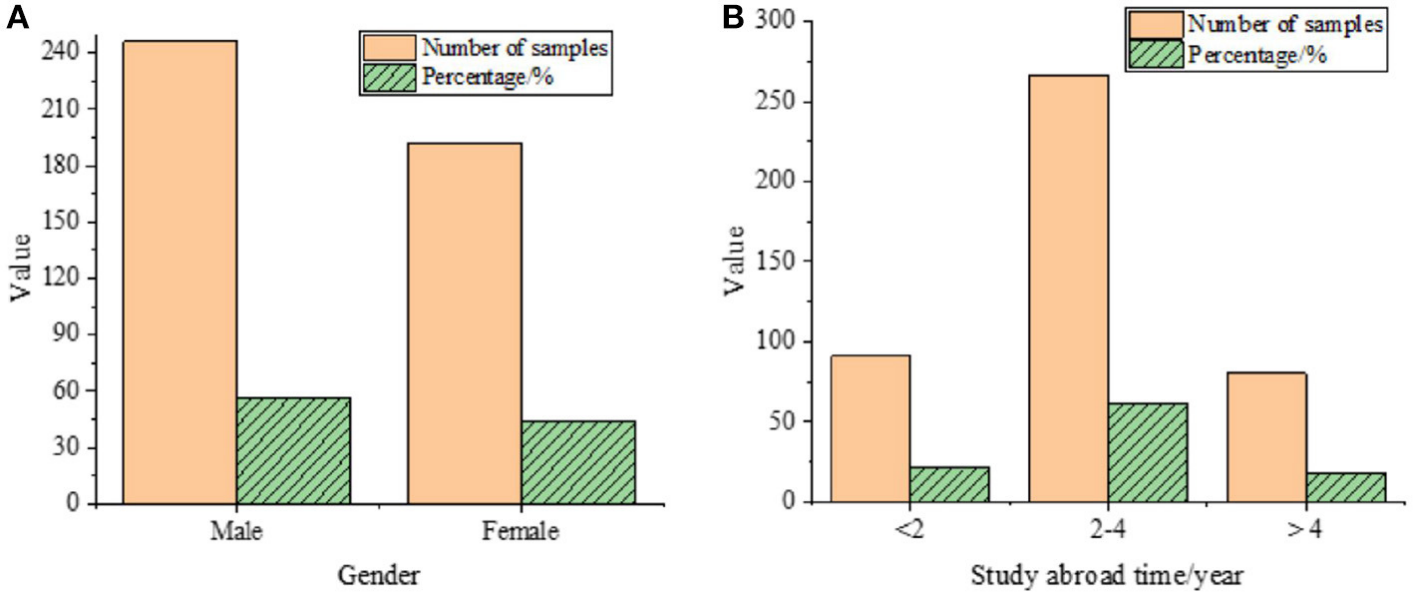
Descriptive statistics of sample demographic features. **(A)** Gender, **(B)** Duration of study abroad.

Subsequently, for a more intuitive understanding of the QS, Figure 2 statistically analyzes the samples' demographic features, such as gender distribution and duration of study abroad.

As depicted in Figure 2, according to the basic statistics description of 438 respondents, respondents studying abroad for 2-4 years account for the most, 60.9%. In terms of gender distribution, males and females account for 56% and 44%, respectively, relatively uniform.

### Reliability Test of Questionnaire

**Figure 3:**
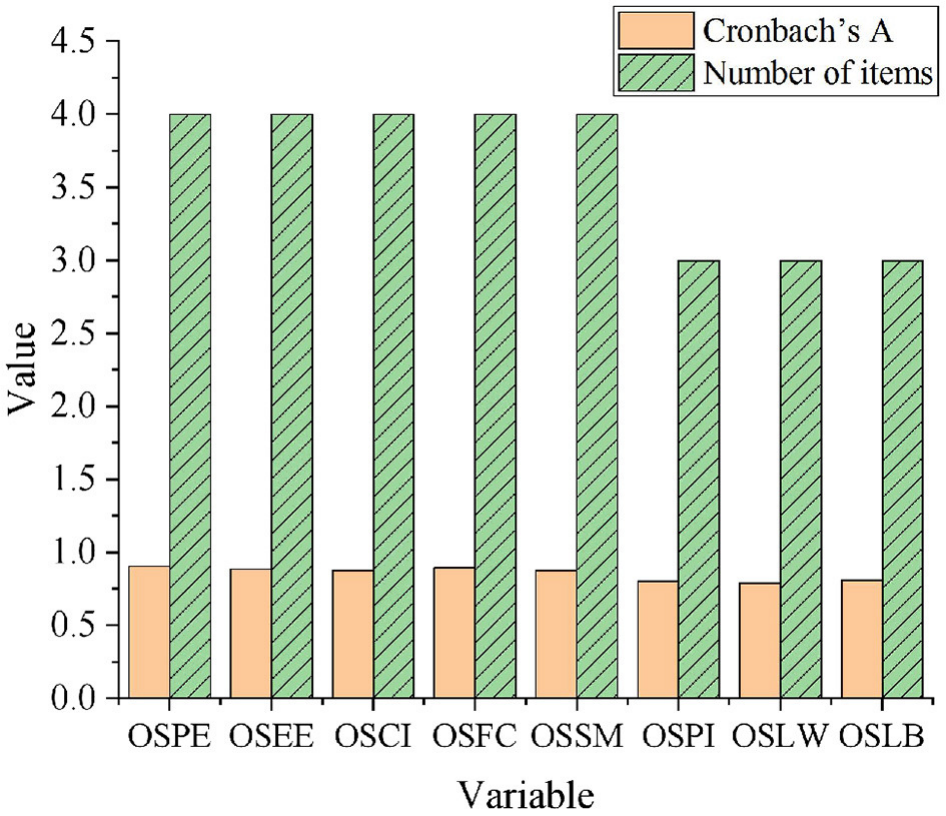
Reliability of each dimension.

For the reliability of questionnaire samples, Cronbach's alpha coefficient needs to be used for reliability evaluation, as shown in Figure 3.

### Sample Reliability Analysis

The sample reliability analysis employs Cronbach's Alpha coefficient for evaluation, as outlined in Figure 3.

Figure 3 suggests that the Cronbach's α coefficients of OSPE, OSEE, OSCI, OSFC, and OSSM are 0.902, 0.884, 0.878, 0.891, and 0.876, respectively. The Cronbach's α coefficients of OSPI, OSLW, and OSLB are 0.801, 0.789, and 0.808. Overall, the α coefficients of these eight dimensions of Cronbach are greater than the standard value of 0.7. Therefore, the collected QS data has good internal reliability and can be further studied.

### Sample Validity Analysis of Samples

#### Questionnaire Validity Test

**Figure 4:**
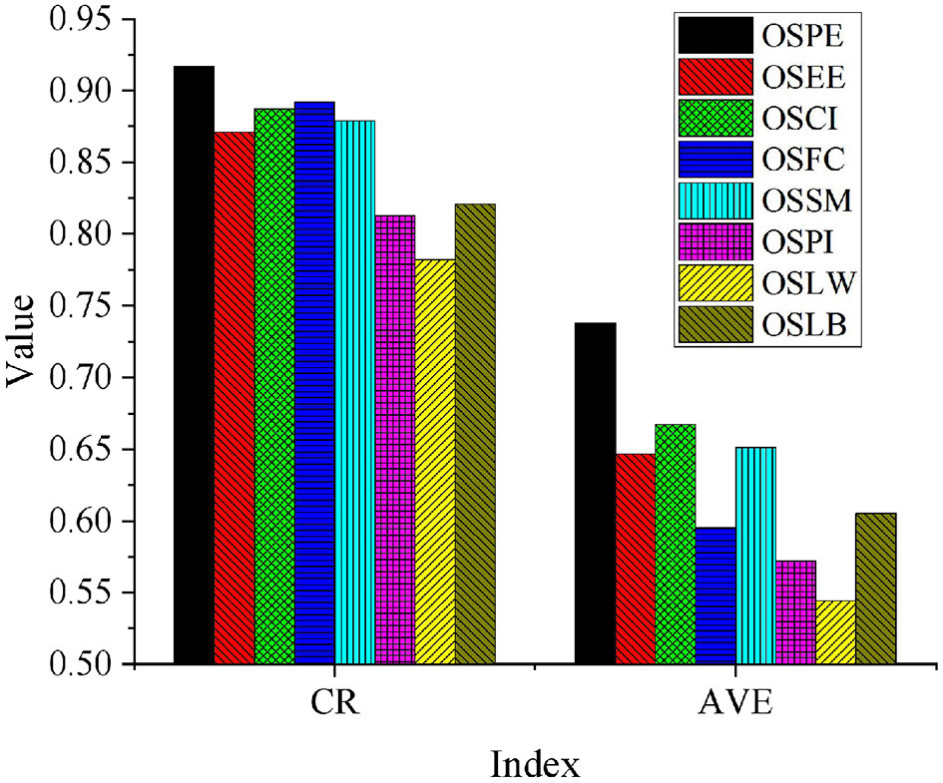
Convergent validity of the scale.

Then, Amos 23.0 is used to extract Composite Reliability (CR) and Average Variance (AVE) to analyze the convergence validity of the questionnaire. The measurement results are shown in Figure 4.

As Figure 4 presents, CR of OSPE, OSEE, OSCI, OSFC, OSSM, OSPI, OSLW, and OSLB is greater than 0.7. AVE is greater than 0.5, indicating that the measurement scale has good internal consistency. These eight potential variables obtained relatively high changes from the corresponding observed variables. Therefore, the correlation is relatively high, indicating that the validity of the questionnaire has been tested.

In addition to testing the CR and AVE, it is also necessary to test the Discriminant Validity (DV). Specifically, DV refers to the degree of difference between potential variables. Table 3 lists the DV results of the design scale.

**Table 3:** DV of the scale.

	**OSPE**	**OSEE**	**OSCI**	**OSFC**	**OSSM**	**OSPI**	**OSLW**	**OSLB**
OSPE	0.874							
OSEE	0.401^**^	0.815						
OSCI	0.474^**^	0.374^**^	0.832					
OSFC	0.542^**^	0.388^**^	0.451^**^	0.782				
OSSM	0.399^**^	0.402^**^	0.366^**^	0.482^**^	0.823			
OSPI	0.443^**^	0.353^**^	0.400^**^	0.620^**^	0.481^**^	0.771		
OSLW	0.633^**^	0.578^**^	0.588^**^	0.673^**^	0.619^**^	0.654^**^	0.749	
OSLB	0.429^**^	0.313^**^	0.406^**^	0.632^**^	0.531^**^	0.512^**^	0.644^**^	0.788

*The AVE open root of each construct is on the main diagonal and below the diagonal is the Pearson correlation coefficient between each construct.*

** *indicates the pearson correlation coefficient between each structure is significant*.

As detailed in Table 3, the diagonal values are greater than their corresponding rows and columns, which shows that the measurement scale has good DV. In conclusion, the reliability and validity of the designed measurement scale are relatively good, based on which the subsequent analysis can be carried out.

### SEM Test

#### Model Fitting Evaluation

**Figure 5:**
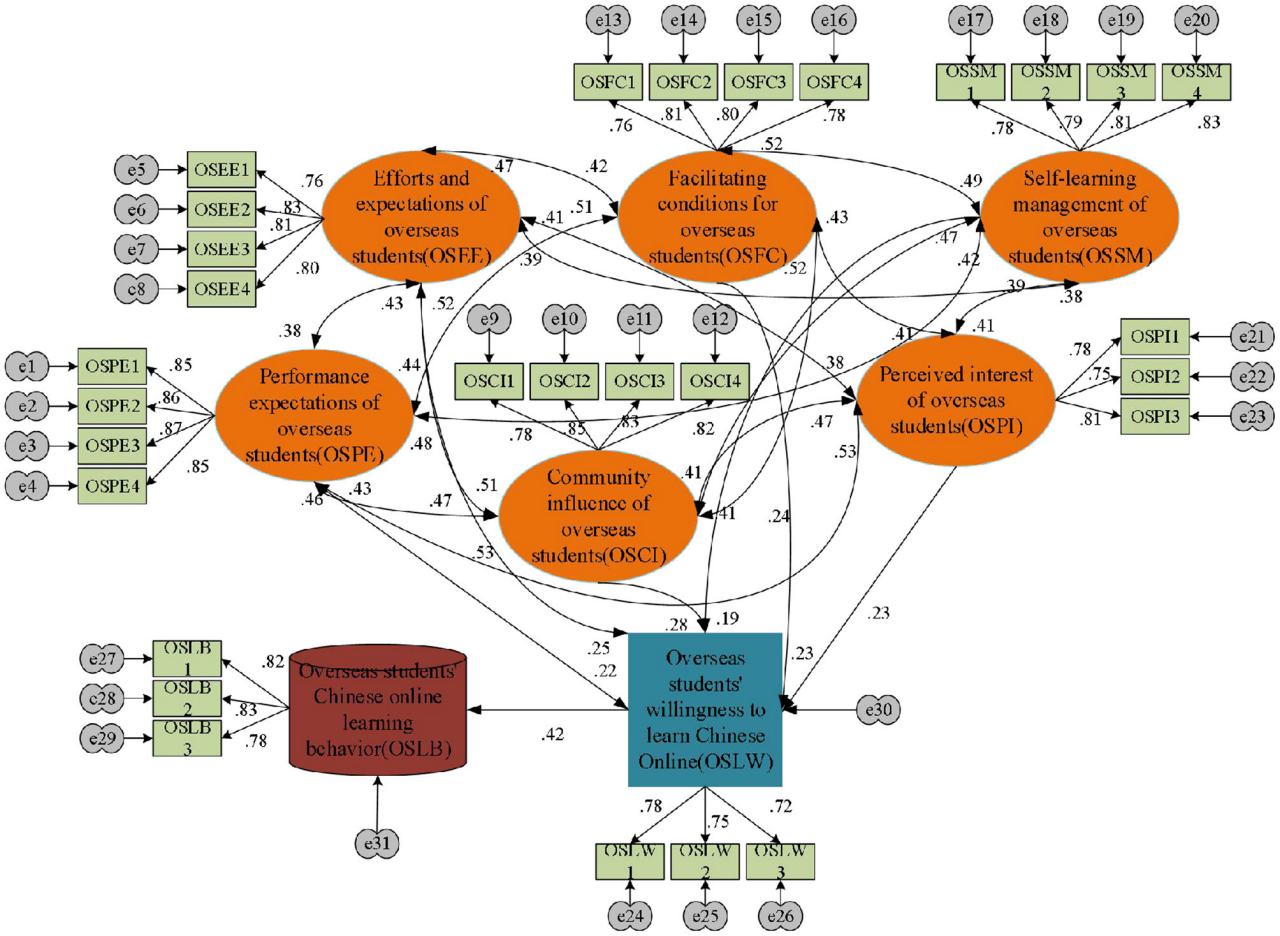
Standardized path coefficient of the initial model.

SEM is introduced into SPSS, and the parameters are estimated by the maximum likelihood estimation method. Table 4 and Figure 5 show standardized estimation results and non-standardized estimation results, respectively.

**Table 4:** Fitting results of initial SEM.

**Fitting index**
**CMIN/DF**	**RMR**	**GFI**	**AGFI**	**NFI**	**IFI**	**TLI**	**CFI**	**RMSEA**
1.50	0.038	0.910	0.903	0.925	0.923	0.959	0.968	0.032

Figure 5 shows the standardized path coefficient structure of the initial model. In the model, the management, expectation, and community influence of foreign students are comprehensively analyzed, and a unified initial parameter model is implemented through the standardization of coefficients. Additionally, for the fitting index between the standardized fitting result of the model and the initial model, the data arrangement is shown in Table 4.

Table 4 manifests the fitting index of the initial model.

As displayed in Table 4, among all fitting indexes, CMIN/DF is less than 0.3, RMR is less than 0.5, RMSEA is less than 0.08, and the values of other fitting indexes are greater than 0.9. Thus, the fitting indexes have reached the ideal value, and there is no need to modify the model.

#### Path Analysis

**Figure 6:**
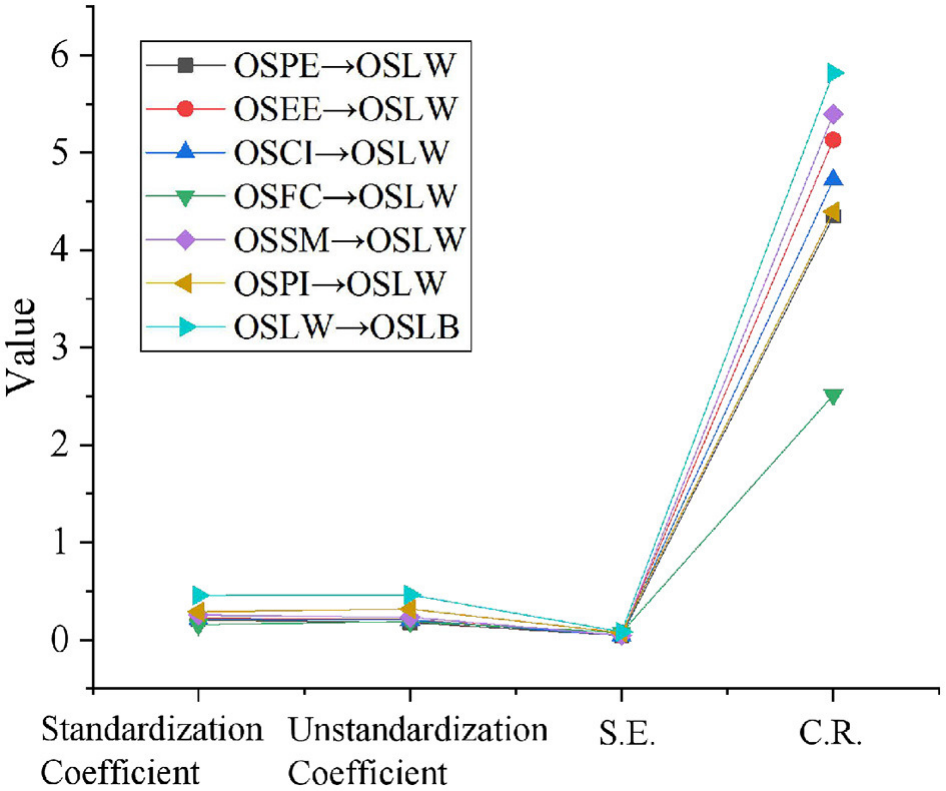
Corrected path analysis results.

Figure 6 analyzes the path of the modified model using Amos 23.0. OSPE->OSLW, OSEE->OSLW, OSCI->OSLW, OSFC->OSLW, OSSM->OSLW, OSPI->OSLW represent the impact degree of OSPE, OSEE, OSCI, OSFC, OSSM, and OSPI on OSLW. And OSLW->OSLB suggests the impact degree of OSLW on OSLB.

As indicated in Figure 6, when P <0.001, the standardization coefficients of the impact of OSPE, OSEE, OSCI, OSFC, OSSM, and OSPI on OSLW for OCL is 0.203, 0.224, 0.211, 0.156, 0.254, and 0.289, respectively. The standardized coefficient of the impact of OSLW for OCL on OSLB is 0.457. Therefore, research hypotheses hold.

### Research Hypothesis Test

Table 5 reveals the test result of the proposed hypotheses.

**Table 5:** Hypothesis test results.

**Label**	**Research hypotheses**	**Verified or falsified**
H1	OSPE has a significant positive impact on OSLW for OCL.	Verified
H2	OSEE has a significant positive impact on OSLW for OCL.	Verified
H3	OSCI has a significant positive impact on OSLW for OCL.	Verified
H4	OSFC has a significant positive impact on OSLW for OCL.	Verified
H5	SLM has a significant positive impact on OSLW for OCL.	Verified
H6	PI has a significant positive impact on OSLW for OCL.	Verified
H7	BI has a significant positive impact on OSLB for OCL.	Verified

According to Table 5, all hypotheses (H1, H2, H3, H4, H5, H6, and H7) proposed have been well verified. OSPE, OSEE, OSCI, OSFC, OSSM, and OSPI all have a direct positive impact on OSLW.

According to the research and analysis, OSPE, OSEE, OSCI, OSSM, and OSPI have a significant positive impact on OSLW for OCL and indirectly affect OSLB through OSLW. The results show that OSs can improve their WTL for OCL by improving OSPE. Additionally, OSs feel that the lower the difficulty of OCL is, the stronger their WTL is. The OSFC has a positive impact on OSLW and OSLB for OCL. At present, learners' OSFC perception is relatively low, which may be because they used to learn Chinese offline, which is now hindered by the impact of the COVID-19 epidemic. Therefore, OSs have not fully understood OCL and have little perception of the facilitating conditions. Moreover, OSs' online learning might get distracted because they might have difficulties in understanding, so autonomy and self-discipline have become important factors affecting OSs' OCL. Personal interests and hobbies are the main factors affecting OSs' OCL.

## Conclusion

The work adopts the empirical research method based on online WTL research and specific situations. Meanwhile, the work implements the influencing factor model of online education for foreign students, determines six main factors, and compiles a questionnaire. Finally, the initial model is empirically tested according to the sample data. The empirical results show that the Cronbach α coefficients of OSPI, OSLW and OSLB are 0.801, 0.789, and 0.808, respectively. CR is greater than 0.7 and AVE is greater than 0.5, indicating that the measurement scale has good internal consistency. In addition, the standardization coefficients of OSFC, OSSM, and OSPI on OSLW are 0.156, 0.254 and 0.289 respectively, and the standardization coefficient of OCL and OSLW on OSLB is 0.457. Therefore, the research hypothesis holds. It verifies the four core elements of the model and the impact of new factors on the degree of learning intention, and the applicability of the proposed model. Moreover, it enriches the research on variables in the theory of learning intention, and expands the application of the model in OTL. This study provides a reference basis for formulating OTL strategies for foreign students' Chinese curriculum. However, it also has some limitations. Although the work is mainly aimed at the theoretical model based on OSs, due to the diversity and complexity of learners, different learners have differences in cognitive level and learning style. Thus, in the future research, the individual characteristics of learners should be emphasized and the explanatory power of the model should be improved through the specific analysis of individual characteristics. In addition, COVID-19 education has entered a rapid development stage due to the restriction of teaching places. In the follow-up research, the broad development prospects of international students' online education should be focused on.

## Data Availability Statement

The original contributions presented in the study are included in the article/supplementary material, further inquiries can be directed to the corresponding author/s.

## Author Contributions

XZ: editing, data curation and writing—original draft preparation.

## Conflict of Interest

The author declares that the research was conducted in the absence of any commercial or financial relationships that could be construed as a potential conflict of interest.

## Publisher's Note

All claims expressed in this article are solely those of the authors and do not necessarily represent those of their affiliated organizations, or those of the publisher, the editors and the reviewers. Any product that may be evaluated in this article, or claim that may be made by its manufacturer, is not guaranteed or endorsed by the publisher.
